# Bisphenol A (BPA)-Induced Changes in the Number of Serotonin-Positive Cells in the Mucosal Layer of Porcine Small Intestine—the Preliminary Studies

**DOI:** 10.3390/ijms21031079

**Published:** 2020-02-06

**Authors:** Slawomir Gonkowski

**Affiliations:** Department of Clinical Physiology, Faculty of Veterinary Medicine, University of Warmia and Mazury in Olsztyn, Oczapowski Str. 13, 10-718 Olsztyn, Poland; slawomir.gonkowski@uwm.edu.pl

**Keywords:** bisphenol A, serotonin, small intestine, pig

## Abstract

Bisphenol A (BPA) is a substance used in the production of plastics which has a negative impact on many internal organs. Because BPA is normally toxic for the gastrointestinal (GI) tract, the intestine is especially vulnerable to the adverse effects of this substance. The aim of this investigation was to study the influence of two doses of BPA (0.05 mg and 0.5 mg/kg body weight/day) on the number of mucosal cells in the porcine small intestine and containing serotonin (5-hydroxytryptamine, 5-HT). During the experiment, it was demonstrated that both applied BPA doses caused an increase in the number of 5-HT-positive cells located in the mucosal layer of the duodenum, jejunum, and ileum. These changes may be connected with the direct impact of BPA on the intestinal mucosa, the pro-inflammatory and immunomodulatory properties of this substance, and/or the influence of BPA on the neurochemical characterization of nervous structures supplying the intestine.

## 1. Introduction

Bisphenol A (BPA) is commonly used in the manufacture of plastics and is, therefore, present in numerous everyday objects including household goods, office supplies, car equipment, toys, thermal paper, and more [1]. The most dangerous among these are goods containing BPA which come into contact with drinking water and food, enabling the BPA to transfer or leach from the plastic into the food chain [1]. It is known that BPA arrives in living organisms through the digestive tract, skin or lungs [1]. In light of previous studies, it is also known that BPA may impair the functioning of internal organs and systems, including (among others) the nervous, gastrointestinal, cardiovascular, reproductive, and excretory systems [1,2]. Moreover, some investigations have shown a correlation between the degree of exposure to BPA and the risk of diabetes, heart attack, neoplasms, and even autism [1,2,3,4]. 

Because the main path of toxic infiltration by BPA is via the digestive tract, the stomach and intestine are especially vulnerable to the adverse effects of this substance. The previous studies have indicated that BPA damages the intestinal barrier function and may change nociception in the gastrointestinal tract [5,6]. Moreover, BPA promotes inflammatory processes in the stomach and intestine [7,8]. It is also known that even relatively low doses of BPA can cause changes in the neurochemical characterization of nervous structures supplying the gastrointestinal tract [9].

Nevertheless, many of the aspects connected with the influence of BPA on the intestine remain unclear. One of these is the influence of BPA on serotonin (5-hydroxytryptamine, 5-HT)-positive cells in the intestinal mucosal layer, and the function of 5-HT in processes connected with the harmful effects of BPA. 

5-HT is a biogenic amine that may act as a neurotransmitter and local hormone and is involved in various physiological mechanisms [10]. It should be pointed out that about 95% of the serotonin in the body is located in the GI tract (the rest being located in the brain), and the majority of gastrointestinal serotonin is found in the enterochromaffin cells (ECs) in the gastrointestinal mucosa [11]. It is known that the 5-HT in the GI tract is involved in activation of gut motility, participation in sensory and pain stimuli conduction, and in the regulation of the intestinal secretions [10,11]. Moreover, previous studies have reported the important roles of 5-HT in pathological processes occurring in the GI tract, especially in inflammatory bowel disease, ulcerative colitis, and Crohn’s disease [11,12], and that 5-HT may be a factor participating in stimulation of nausea and vomiting as well as the mechanisms leading to diarrhea [11,12,13,14]. 

Current knowledge about the correlation between exposure to BPA and levels of 5-HT is relatively scarce and is limited to the central nervous system [15,16]. The influence of BPA on 5-HT-positive cells in the gastrointestinal tract has not been studied at all and, therefore, the aim of the present investigation was to study the influence of various doses of BPA on the number of 5-HT-positive cells in the mucosal layer within the small intestine of the domestic pig which, due to the similarities in anatomical and biochemical organization of the GI tract to man, is considered as a good animal model for investigations of phenomena occurring in the human GI tract [17].

## 2. Results

**Figure 1:**
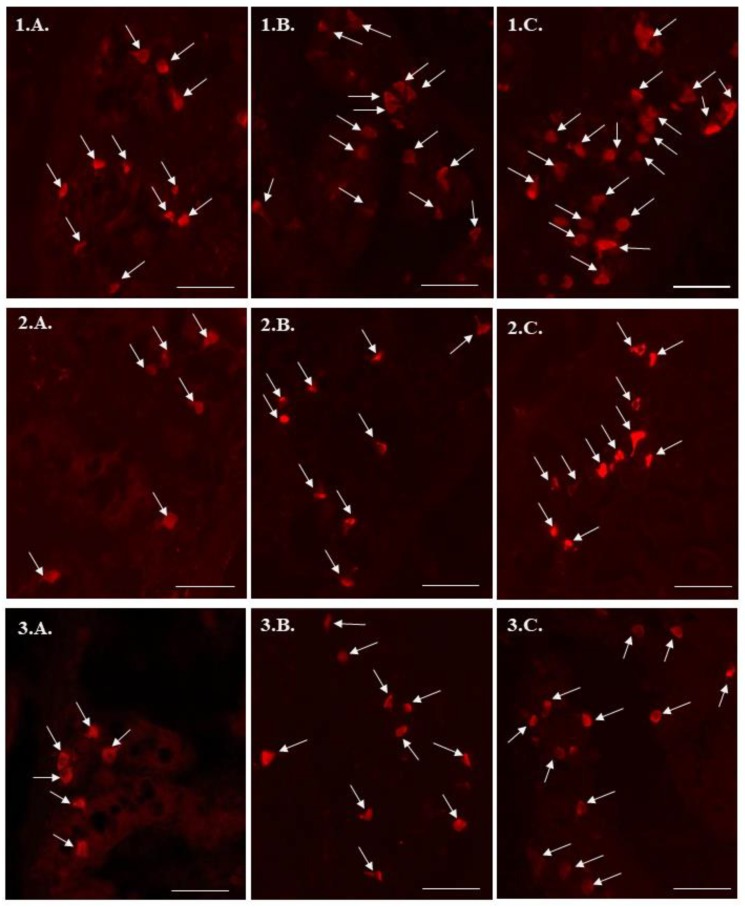
Serotonin – immunoreactive cells (indicated with arrows) in the mucosal layer of the porcine duodenum (**1**), jejunum (**2**) and ileum (**3**) in control animals (**A**), and animals treated with low (**B**) and high (**C**) dose of bisphenol A. Scale bar 50 μm.

5-HT-positive cells were observed in the mucosal layer of all segments of the small intestine, both under physiological conditions as well as under the impact of BPA (Table 1, Figure 1). In control animals, the largest number of such cells was noted in the duodenum (Figure 1(1.A)), where the average number of 5-HT-immunoreactive cells amounted to 12.48 ± 0.53 cells per microscopic observation field. In the jejunum (Figure 1(2.A)) and ileum (Figure 1(3.A)), the number of cells immunoreactive to 5-HT was significantly lower at 7.47 ± 0.22 in the jejunum and 8.59 ± 0.30 in the ileum (Table 1).

Both doses of BPA used in this study caused an increase in the number of 5-HT-immunoreactive cells in the mucosal layer of all segments of the small intestine. Under low doses of BPA (0.05 mg/kg body weight/day,) the most visible changes were in the duodenum (Figure 1(1.B)), where the average number of 5-HT-positive cells amounted to 16.26 ± 0.70 per observation field (Table 1). Slightly fewer changes were noted in the jejunum (Figure 1(2.B)) and ileum (Figure 1(3.B)), where the number of mucosal cells immunoreactive to 5-HT was 10.08 ± 0.17 and 12.11 ± 0.61, respectively (Table 1).

In animals treated with high doses of BPA (0.5 mg/kg body weight/day), the most visible changes were also noted in the duodenum (Figure 1(1.C)). In this section of the small intestine, the average number of 5-HT-positive cells in the mucosal layer amounted to 20.21 ± 0.10 cells per observation field (Table 1). In the jejunum (Figure 1(2.C)) and ileum (Figure 1(3.C)), the number of such cells in animals receiving the higher dose of BPA reached 13.46 ± 0.31 and 14.69 ± 0.61, respectively (Table 1).

All results obtained from the particular animals and average values are presented in Table 1.

## 3. Discussion

The obtained results show that BPA may change the number of 5-HT-positive cells in the mucosal layer of the porcine small intestine. It should be emphasized that the lower dose of BPA used in this experiment (0.05 mg/kg body weight/day) is recommended as a tolerable daily intake (TDI) or reference dose in some countries [18]. The present study has indicated that such a dose is, however, not neutral for living organisms, in accordance with the previous studies in which the influence of this dose of BPA on the innervation of the GI tract and uterus was also observed [9,19].

The observed changes probably result from the direct impact of BPA on the intestinal mucosa. It is known that BPA absorption results in intensification of apoptosis and inhibition of proliferation of intestinal epithelial cells [20] and contribute to a reduction in mucin secretion from the intestinal mucosal cells [6]. Through the above-mentioned activity, BPA causes the disruption of the intestinal barrier and may lead to an increase in intestinal permeability [21]. 

These influences of BPA on the mucosal layer may be the reason for the increase in the number of 5-HT-positive mucosal cells noted in the present study, because it is known that 5-HT is a factor supporting the integrity of the intestinal mucosal layer and stimulating fluid and mucus secretion as well as ions transport, which processes are important for mucosal protection [10,11]. 

The next probable reason for the observed changes in the relatively well-known pro-inflammatory activity of BPA and its effects on the immune system [22]. This is all the more likely considering that previous studies have shown both the participation of intestinal 5-HT in inflammatory processes in the GI tract and its involvement in the modification of immune responses [11]. Participation of intestinal 5-HT in inflammatory mechanisms has been confirmed by previous studies, in which the increase of 5-HT levels in the intestinal mucosa have been observed during “natural” inflammatory processes in humans (for example, inflammatory bowel disease, Crohn’s disease, and ulcerative colitis) and various types of experimental inflammation in animals [11,12]. On the other hand, the doses of BPA used in this experiment cause somewhat unclear histopathological changes limited to the ileum [9]. However, it cannot be excluded that the increase in the number of 5-HT-positive cells noted in the present study is the first subclinical sign of the pro-inflammatory activity of BPA.

Moreover, observed changes may be connected with the fact that even low doses of BPA might affect the neurochemical characterization of the nervous structures in the enteric nervous system [9]. Due to interactions between enterochromaffin cells and the nervous system [23], the fluctuation in neuronal active substances in the enteric neurons may result in changes to the number of cells containing 5-HT. It is more likely that 5-HT produced by the enterochromaffin cells in the intestinal mucosa may act on the intrinsic primary afferent neurons, which in turn affect motor neurons located in the intestine wall [11,12], the result of which is the influence of 5-HT on intestinal muscles activity. The changes noted in the present study may be connected with these mechanisms, because BPA is a factor which it seems likely may decrease intestinal motility [24].

To sum up, the obtained results indicate that BPA increases the number of 5-HT-positive cells in the porcine small intestine, which is most likely to be connected with the direct impact of BPA on the intestinal mucosal layer and/or the pro-inflammatory properties of this substance. In turn, clinically speaking, the obtained results suggest that even exposure to low doses of BPA can foster pathological processes in the GI tract. However, the mechanisms of changes noted in the present study are not clear under the current state of knowledge. On the one hand, they may result from an increase in the synthesis of 5-HT under the impact of BPA, which aims to maintain homeostasis in the GI tract and reduce and/or eliminate the effects of BPA action. On the other hand, the changes may be caused by the inhibition of serotonin release from the enterochromaffin cells by BPA. So, establishing all the aspects connected with the mechanisms of participation of serotonin in the GI tract resulting from exposure to BPA certainly requires further comprehensive studies.

## 4. Materials and Methods

The study was performed on 15 female pigs of the Piétrain x Duroc breed at the age of 8 weeks. All experimental activities received approval from the Local Ethical Committee for Experiments on Animals in Olsztyn (Poland) (decision numbers 28/2013 of 22 May 2013 and 65/2013/DLZ of 27 November 2013).

After an adaptive period (five days), the animals were randomly divided into three groups, one control and two experimental groups (five animals in each group). Control animals (C group) received empty capsules during morning foraging for twenty-eight days. In the experimental groups, BPA in capsules was given as follows: pigs of experimental group I (Exp I G) received BPA in 0,05 mg/kg body weight/day doses and animals in experimental group II (Exp II G) were treated with BPA in 0.5 mg/kg body weight/day doses. The method and time of BPA administration were the same as for the administration of empty capsules in the control animals.

After 28 days, all the animals were euthanized. Immediately after this, fragments (about 3 cm in length) of various parts of the small intestine were collected as follows: duodenum located about 3 cm after the gastric pylorus, ileum located about 50 cm after the gastric pylorus, and ileum located 3 cm before the ileocecal valve.

Immediately after collecting the intestinal fragments, they were fixed in 4% buffered paraformaldehyde (pH 7.4) for 1 h. The tissues were then rinsed in phosphate buffer for three days and stored in 18% phosphate-buffered sucrose at 40 °C for at least three weeks. After this period, the fragments of the intestine were frozen at −200 °C, cut perpendicular to the gut lumen into 10 μm-thick sections with a microtome (HM 525, Microm International, Walldorf, Germany), and mounted on microscope slides.

The routine single-labelling immunofluorescence technique was used to evaluate the number of 5-HT-positive cells in the mucosal layer of the particular segments of the intestine. For this purpose, the slices of the intestine were dried at room temperature (rt) for 45 min. and incubated with a blocking solution (10% normal goat serum, 0.1% bovine serum albumin, 0.01% NaN3, Triton x-100 and thimerosal in PBS) for 45 min. After this period, fragments of intestine were incubated overnight; rt, in a humid chamber with antiserum against serotonin (rabbit anti-5-HT antibody, Zymed Laboratories, San Francisco, CA, USA, lot no. 30778610 R, working dilution 1:1000). The next day, slides with intestinal slices were incubated (rt, in a humid chamber) with species-specific secondary antiserum conjugated with fluorochrome Alexa fluor 546 (AF 546 goat anti-rabbit IgG (H + L) ThermoFisher Scientific, Waltham, MA, USA, working dilution 1:1000) for 1 h and closed with buffered glycerol and closed slides. During the study, typical standard controls of labeling specificity, including pre-absorption, omission, and replacement tests were performed.

The labeled fragments of the intestine were evaluated with an Olympus BX51 microscope equipped with epi-fluorescence and appropriate filter sets. The evaluation of the number of 5-HT-positive cells in the mucosal layer of the particular fragments of the small intestine was based on the counting of all such cells per the microscopic observation field (0.15 mm^2^). Serotonin-positive cells were counted in five slices of the particular segments of the small intestine, in ten randomly selected observation fields (within the mucosal layer) per section. Therefore, 50 observation fields were evaluated in each segment of the small intestine (duodenum, jejunum, and ileum) from each animal. In order to prevent double-counting of the same cells, intestinal slices included in the study were located at least 200 µm apart. The obtained data were pooled and presented as the mean ± the standard error of measurement (SEM).

Statistical analysis was performed using univariate ANOVA test (GraphPad Prism v. 3.0, GraphPad Software Inc., San Diego, CA, USA). The differences were considered statistically significant at *p* ≤ 0.05.

## Figures and Tables

**Table 1:** The average number of serotonin-positive cells (per microscopic observation field—0.15 mm^2^) in the mucosal layer of the porcine small intestine in control animals (C group) and in animals treated with low (Exp I G) and high (Exp II G) doses of bisphenol A.

Part of the Intestine	C Group	Exp I G	Exp II G
**Duodenum**			
Animal 1	13.4	15.26	22.76
Animal 2	13.96	16.34	21.72
Animal 3	11.6	18.42	20.33
Animal 4	12.26	16.94	17.34
Animal 5	11.16	14.34	18.92
Average ± SEM	12.48 ± 0.53	16.26 ± 0.70 *	20.21 ± 0.10 *
**Jejunum**			
Animal 1	7.04	9.76	13.88
Animal 2	6.96	10.34	12.96
Animal 3	7.54	10.18	12.56
Animal 4	7.64	10.52	13.64
Animal 5	8.15	9.6	14.24
Average ± SEM	7.47 ± 0.22	10.08 ± 0.17 *	13.46 ± 0.31 *
**Ileum**			
Animal 1	8.42	13	13.92
Animal 2	8.38	12.14	15.58
Animal 3	9.64	11.16	13.06
Animal 4	7.78	10.42	14.36
Animal 5	8.72	13.84	16.52
Average ± SEM	8.59 ± 0.30	12.11 ± 0.61 *	14.69 ± 0.61 *

Statistically significant differences (*p* ≤ 0.05) between the C group and Exp I group, as well as between C group and Exp II group in particular segments of the intestine are marked with *.
